# Prenatal Internal Locus of Control Is Positively Associated with Offspring IQ, Mediated through Parenting Behavior, Prenatal Lifestyle and Social Circumstances

**DOI:** 10.3389/fpsyg.2017.01429

**Published:** 2017-08-22

**Authors:** Jean Golding, Steven Gregory, Genette L. Ellis, Yasmin Iles-Caven, Stephen Nowicki

**Affiliations:** ^1^Paediatric and Perinatal Epidemiology, Centre for Child and Adolescent Health, Bristol Medical School, University of Bristol Bristol, United Kingdom; ^2^Department of Psychology, Emory University, Atlanta GA, United States

**Keywords:** ALSPAC, maternal locus of control, child cognition, child IQ, verbal IQ, performance IQ

## Abstract

Locus of control (LOC) is a measure that identifies the likelihood as to whether an individual considers what happens to him is largely a matter of luck or fate (known as externally oriented) or whether it is something that the individual can influence (internality). Here we have used data collected as part of the Avon Longitudinal Study of Parents and Children (ALSPAC) to determine the associations between the mothers’ LOC orientation before the birth of the child and her child’s cognition measured at age 8. Using results from 6801 children we show that maternal internal LOC is associated with increased ability in offspring IQ, as measured using the WISC, with children of internally oriented mothers having an advantage of approximately 7 IQ points at age 8. As a sensitivity analysis we used the IQ test results of a sample of 986 preschool children tested using the WPSSI at age 4. A similar advantage was found among the offspring of the internally oriented mothers. We investigated mechanistic explanations for these results firstly by determining the extent to which three separate sets of factors known to be influenced by the LOC orientation might explain these findings. We showed that (a) perinatal life-style exposures, (b) parenting attitudes and strategies and (c) socio-economic circumstances, largely explain the mechanism through which the internality of the mother influences the cognition of the child. Similar effects were found using the smaller sample tested at age 4. The results indicate that efforts made to foster internality in adolescents and young adults prior to parenthood may result in improvements in the cognitive development of the next generation. Intervention studies are urgently needed.

## Introduction

### The Importance of Locus of Control

The purpose of the present study was to examine the possibility that prenatal maternal locus of control (LOC) was associated with children’s IQ outcomes and, if so, to identify possible mechanisms. The concept of LOC was introduced to psychology by Rotter (1966) who defined it as follows: “Internal vs. external control refers to the degree to which persons expect that a reinforcement or an outcome of their behavior is contingent on their own behavior or personal characteristics vs. the degree to which persons expect that the reinforcement or outcome is a function of chance, luck, fate, is under the control of powerful others, or is simply unpredictable. Such expectancies may generalize along a gradient based on the degree of semantic similarity of the situational cues.”

Since its introduction as a psychological construct, LOC has been the object of over 17,000 studies that have shown it to be related to a variety of personal and social outcomes (Nowicki and Duke, 1982, 2016; Nowicki, 2016). Its introduction into the psychological literature has most often been tied to cognitive and educational outcomes. In perhaps the largest and most influential study of its kind in the 1960s, Coleman et al. (1966) assessed nearly half a million students and found that LOC was the most significant predictor of educational outcomes in African American students and second most significant in European American students.

Reviews of LOC × academic achievement studies have shown internality to be associated with higher average grades and achievement test scores (Findley and Cooper, 1983; Kalechstein and Nowicki, 1997). In some instances, the LOC achievement association remained significant even when IQ scores were controlled for (Ollendick and Ollendick, 1976; Brown, 1980; Finch et al., 1988). Past research suggests that LOC orientation precedes academic achievement rather than vice versa (Stipek, 1980).

Surprisingly, research regarding the association between LOC and IQ is sparse. More often, studies have looked at the association between child IQ and child LOC with child and adult outcomes involving academic performance or educational attainment of some sort (Von Stumm et al., 2009). Aside from parent child rearing attitudes (Osborn and Milbank, 1987; Von Stumm et al., 2009; Wickline et al., 2011), rarely have parent personality factors like LOC, been examined for their possible association with children’s IQ at different developmental times. One exception is Flouri (2006) who found that mothers’ interest in their children’s education and child internal LOC, predicted later higher educational attainment; mothers’ LOC, however, was not assessed.

If a maternal personality factor like LOC was found to be significantly associated with child IQ outcomes, it offers the intriguing possibility that changing mother’s LOC might be associated with a change in the expression of IQ in children across time. Of course, association is not causation, but it does suggest a place to look. Support for a possible parent LOC, child IQ outcome association comes from our previous research which showed that prenatal maternal internal LOC was associated with more positive personal and social outcomes in their children as early as age 4 weeks and as late as age 11 (Nowicki et al., 2017; Nowicki et al., submitted).

Of specific relevance to the present study, change in maternal LOC toward internality over the child’s first 6 years was found to be associated with more positive teacher rated personality characteristics for children at age 8 and 11 years of age when compared to children of mothers who either remained or become externally controlled over the same time-period (Nowicki et al., in preparation). The authors argued that this was consistent with what can be expected theoretically and empirically. Thus compared to external mothers, internal mothers are likely to be more consistent and contingently responsive to their children’s behavior, provide more stimulating family environments, emphasize earlier independence training, more often engage in granting autonomy, use less hostile and more educative disciplinary techniques and provide a warmer, emotionally secure learning environment (Carton and Nowicki, 1996; Schneewind, 1997; Wickline et al., 2011; Nowicki, 2016). These are all factors that would be likely to facilitate positively the expression of intellectual capabilities in their children.

### The Present Study

The purpose of the present study was to examine the possibility that mothers’ LOC measured during pregnancy would be associated with the IQ of their children at 8 years of age. It is hypothesized that, because theoretically, internality in mothers should be associated with the kinds of parenting behaviors that facilitate the expression of intelligence, it will be significantly related to higher IQ measured in children compared to children of prenatally external mothers. In addition, to assess whether the association is mediated by other factors that are linked to LOC orientation we considered parent attitudes, perinatal exposures to the fetus/infant including smoking and breast feeding, and social economic status. Supporting evidence will be sought using the IQ test results of a sub-group of the cohort at age 4.

## Materials and Methods

### The Design of the ALSPAC Study

Avon Longitudinal Study of Parents and Children (ALSPAC) is a pre-birth cohort designed to determine the environmental and genetic factors that are associated with health and development of the study offspring (Golding and ALSPAC Study Team, 2004; Boyd et al., 2013). It recruited 14,541 pregnant women resident in Avon, United Kingdom with expected dates of delivery between April 1st, 1991 and December 31st, 1992 (an estimated 80% of the eligible population). Data were collected at various time-points using self-completion questionnaires, biological samples, hands-on measurements, and linkage to other data sets. For full details of all the data collected see the study website: www.bristol.ac.uk/alspac/researchers/data-access/data-dictionary/.

Ethical approval for the study was obtained from the ALSPAC Ethics and Law Committee and the Local Research Ethics Committees (LRECS). They agreed that consent was implied if questionnaires were returned. Informed written consent was obtained for all biological samples prior to analysis, and for certain invasive procedures during the optional hands-on assessments.

With advice from the ALSPAC Ethics and Law Advisory Committee it was decided not to enroll the study fathers directly, but rather to send to the mother a questionnaire for her partner and ask her if she would like her partner to be involved. If she wished she could give the questionnaire to him (or her) with a separate reply-paid envelope for return. As the study deliberately chose not to record whether or not the mother had invited her partner to complete a questionnaire, it was unable to send reminders to the partners themselves. During pregnancy questionnaires were returned by 76% of partners of women who were taking part in the study.

As part of the study design, there was a concerted effort before the child’s birth to obtain from the parents details of their personalities, moods and attitudes, including a measure of their LOC. The pregnant women were sent four questionnaires during the pregnancy, one of which contained the LOC scale; in parallel they were sent two questionnaires for their partners to complete.

### The LOC Measure

The LOC measure used in the present study is a shortened version of the adult version of the Nowicki-Strickland Internal-External LOC scale (ANSIE). The ANSIE (Nowicki and Duke, 1974) comprises 40 items in a yes/no format, which assess perceived control. This measure was chosen over other scales more specifically related to perceived control over health, as it was considered that this more generalized scale would relate to other factors in addition to health outcomes. Construct validity for the scale has been found in the results of over a thousand studies (Nowicki, 2016). The version used in the present study comprises 12 of the original 40 items which were chosen after factor analysis of the ANSIE administered as a pilot to 135 mothers. The 12 questions loaded onto a single factor of general LOC. The 12 questions used are shown elsewhere (Golding et al., 2017). From the responses from 12,471 women a ‘LOC score’ was derived, the higher the score the more external the LOC. The scores ranged from 0 to 12. The frequency was normally distributed with a median of 4. For this study, external LOC was defined as having a score of >4. This cut-off identified 45.2% of the women as externally controlled (ELOC).

### Measures of Cognition

#### IQ Testing at Age 8

The WISC-III ^UK^ (Wechsler et al., 1992) was used to assess cognitive function at age 8. At the time, it was the most up to date version of the Wechsler Intelligence Scale for Children, the most widely used individual ability test worldwide. A short form of the measure was employed where alternate items (always starting with item number 1 in the standard form) were used for all subtests, except for the coding subtest which was administered in its full form. Hence the length of the session was reduced and children were less likely to tire [such forms have been used successfully in several studies (Stricker et al., 1968; Finch and Childress, 1975)]. All tests were administered by members of the ALSPAC psychology team. The final WISC IQ scores (verbal, performance and total IQ) were calculated from the total scaled scores as described above using the look-up tables provided in the WISC manual.

#### IQ Testing at Age 4

Cognitive development at age 4 was assessed using the Wechsler Pre-school and Primary Scale of Intelligence or WPPSI (The Psychological Corporation, 1993) on a quasi-randomly selected 10% sample of the whole cohort. This assessment comprises 10 subtests, five verbal and five performance. The verbal subtest scores combine to make up the *verbal IQ* (VIQ), and the performance scores combine to make up the *performance IQ* (PIQ). The ten subtest scores together combine to produce a full-scale IQ score.

If a child completed fewer than four subtests on the performance scale then the performance IQ score could not be calculated (and therefore nor would the full-scale score). If, however, the child completed four out of the five, the mean of the four subtests was calculated and imputed for the subtest not completed, so that a performance score could be computed. This *prorating* is standard WPPSI practice. Identical rules apply for the verbal score.

Every effort was made to ensure inter-rater reliability. The testers were overseen by a tester with long experience of psychometric testing with ALSPAC. He observed each tester, advised them, met with the group regularly to discuss the precise administration of each test, and supervised and checked their scoring. Each tester scored four videos of tests and these were compared.

### Variables Concerned with the Possible Mechanisms

Choice of variables considered as possible mediators concerned those variables which showed both an association with the mother’s LOC and with the child’s IQ as shown in **Table 1**. This demonstrates the associations between the 15 variables which we suspected might be on the causal pathway between the mother’s externality and decreased offspring IQ. In general, the factors that are associated with the mother’s LOC are also associated with the offspring IQ. Thus, the lifestyle, demographic and behavioral factors that are more prominent in the externally oriented mothers are reflected in an increased risk of an IQ < 100 in the offspring; this is particularly apparent for prenatal smoking, low maternal and paternal education level, maternal youth, residence in public housing, and a partner in a manual occupation. Conversely, factors that are less prominent in the externally oriented mothers are reflected in proportionately more children with an IQ ≥ 100 points (e.g., eating oily fish, breast feeding, parenting attitudes and behavior of the mother).

**Table 1:** Possible mediators of offspring IQ at age 8: proportion of children with mothers in each category who are external or internal, and proportion of offspring with IQ < 100 or ≥100.

Potential mediator	Proportions of mothers		Offspring IQ	
	External	Internal	<100	≥100
**Perinatal**				
Mother smoked in pregnancy	27.3%	12.0%	18.4%	11.1%
Mother drank alcohol in pregnancy	47.1%	51.9%	48.4%	54.4%
Mother ate oily fish in pregnancy	49.7%	65.4%	54.7%	66.8%
Mother breast fed baby	68.5%	85.0%	73.5%	86.3%
**Parenting**				
Prenatal disagreement with need for stimulation	22.4%	10.6%	18.0%	10.7%
Mother reads to child 5+ times/week	70.0%	75.7%	72.8%	76.1%
High maternal parenting score	47.0%	53.1%	47.7%	54.9%
Child taken to library by mother	59.4%	72.0%	62.5%	74.7%
Mother sings to child 5+ times/week	70.0%	75.7%	72.8%	76.1%
Child has 10+ books	81.2%	91.2%	84.1%	93.9%
**Demographic**				
Maternal Education < ‘O’level	44.4%	16.6%	34.0%	14.3%
Maternal age < 25	35.0%	19.5%	25.5%	15.2%
Resides in Council accommodation	20.7%	6.6%	13.6%	4.6%
Paternal manual social class	56.1%	34.0%	51.4%	30.5%
Paternal Education < ‘O’level	44.9%	24.8%	42.6%	18.6%

#### The Pre- and Perinatal Chemical Exposures

The following variables were included since there is considerable evidence to implicate these factors in neurocognitive development: (i) mother smoked cigarettes mid-pregnancy (yes/no); (ii) maternal alcohol consumption mid-pregnancy (yes/no); (iii) frequency of maternal consumption of oily fish in third trimester (none/infrequently/at least once a week); (iv) the baby was breast fed (none/any).

#### Parenting Attitudes and Strategies

The parenting attitudes and strategies were based on questions (mostly developed by the ALSPAC team) that the mothers answered in questionnaires completed at home and returned anonymously by post. The factors included were: (i) mothers’ stated attitude during pregnancy to the need of a baby for stimulation (very positive/rest); (ii) the frequency that the mother read to the child at 18 months; (iii) a parenting score derived from the frequency with which the mother attempted to teach and interact with the child at 18 months, the higher the score the more positive the parenting (score ranged from 6 to 51); (iv) frequency with which mother took the child to a library at 42 months (5 point scale of decreasing frequency); (v) number of books child owned (<10/10+) and (vi) the frequency with which the mother sang to the child at 42 months (5-point scale of decreasing frequency).

#### Socio-Economic Circumstances

The variables used were all collected during pregnancy: (i) education level achieved by the mother (5-point scale from very low to University degree); (ii) education level of fathers (similar 5-point scale); (iii) maternal age at the birth of the child (<25; 25–34; 35+); (iv) social class based on the occupation of the father (non-manual/manual); (v) residence in council accommodation (public housing) (yes/no).

### Statistical Approach

This study included all children who took the cognition tests at each age. At age 4, which was designed as a 10% sample of the whole cohort, 1027 children attended the clinic, and 1013, 1016, and 1013 (98.6%), respectively, completed the assessments for verbal, performance and full IQ. At age 8, all eligible children who were still resident in the area were invited. Of the 7488 who attended, 7385 (98.6%), 7377 (98.5%), and 7364 (98.3%) completed the tests to allow calculation of the verbal, performance and total IQ scores. Those children attending the test clinic had a mean age of 103.8 months (*SD* 3.9); 50.1% were boys and 3.9% were non-white; their mean birthweight was 3415 g (*SD* 554) and mean gestation 39.4 (*SD* 1.9) weeks. The mothers had a mean age of 29.1 years and the majority of women resided in owner occupied housing. A comparison of the non-attendees within this population indicated a bias in regard to socio-economic conditions (Supplementary Table 1). As the missing information was not missing at random, we have not used any form of imputation.

The analyses are designed to determine the relationship between the children’s mean IQ scores and the mother’s LOC orientation (comparing those who are external with those who are internal by subtracting the external from the internal). The basic data use an unadjusted multiple regression and note the regression coefficient (*b*), the measure of variance explained (*R*^2^) and the statistical significance (*P*). The analysis was then repeated but taking account of the pre- and perinatal factors (Model A). A separate analysis allowed for the parenting variables (Model B), and a third analysis combined factors A and B. The fourth allowed for socio-demographic factors (Model C). The fifth analysis enabled all the factors in A, B, and C to be taken into account together. Comparison of the regression coefficients and the amount of variance explained for each model was used to deduce the contribution of the different factors in explaining the ways in which the maternal LOC may have impacted on the child’s IQ level.

## Results

### Relationship between Maternal LOC and IQ at 8

The unadjusted difference between the mean IQ of 8-year-old children whose mothers were external compared to those who were internally oriented showed deficits of 7.33, 5.29, and 7.23 IQ points for the verbal, performance and full IQ scales, respectively (**Table 2**); each was highly significant (*P* < 0.0001). It is notable that the maternal LOC only explained 2.27% of *R*^2^ for the performance IQ, whereas almost twice as much of the *R*^2^ was explained for the verbal and full-scale IQ measurements.

**Table 2:** The unadjusted and adjusted associations between the difference between the prenatal maternal internal and external locus of control (LOC) and mean IQ of the offspring at age 8.

Outcome and model	*b* [95% CI]	*n*	*R*^*2*^ %	*P*
**Verbal IQ**				
Unadjusted	7.33 [6.53, 8.13]	6830	4.53	<0.0001
Model A	5.49 [4.63, 6.35]	6094	7.99	<0.0001
Model B	5.47 [4.59, 6.35]	5602	9.68	<0.0001
Model A + B	4.34 [3.43, 5.25]	5341	12.15	<0.0001
Model C	2.35 [1.50, 3.20]	5962	17.00	<0.0001
Model A + B + C	1.90 [0.96, 2.85]	4944	18.43	<0.0001
**Performance IQ**				
Unadjusted	5.29 [4.47, 6.11]	6822	2.27	<0.0001
Model A	3.81 [2.92, 4.70]	6083	4.22	<0.0001
Model B	3.98 [3.07, 4.90]	5591	5.12	<0.0001
Model A + B	3.25 [2.29, 4.21]	5331	6.32	<0.0001
Model C	1.71 [0.78, 2.63]	5955	8.36	0.0005
Model A + B + C	1.36 [0.34, 2.38]	4936	8.95	0.009
**Full-scale IQ**				
Unadjusted	7.23 [6.45, 8.02]	6801	4.56	<0.0001
Model A	5.35 [4.51, 6.19]	6066	8.10	<0.0001
Model B	5.46 [4.60, 6.32]	5575	9.96	<0.0001
Model A + B	4.39 [3.50, 5.28]	5315	12.23	<0.0001
Model C	2.35 [1.51, 3.19]	5938	17.15	<0.0001
Model A + B + C	1.93 [1.00, 2.86]	4922	18.79	<0.0001

*Model A allows for pre- and perinatal exposures; Model B for parenting attitudes and activities; Model C for sociodemographic variables (see text for description of variables). *b* is the regression coefficient*.

To determine how much of the difference was explained by the different behaviors of external compared with internal mothers, we first tested how much the differences were mediated by pre- and perinatal features by comparing the regression coefficients of the unadjusted variable with that derived after allowing for Model A. This showed a drop in the difference for each of the three IQ measures of 1.84, 1.48, and 1.88 points (**Table 2**) or by 25, 28, and 26% of the original regression coefficient, respectively. Each of the four pre-/perinatal variables were independently associated in the model (data not shown).

Model B took the various parenting measures into account with very similar effects (**Table 2**): the reduction in IQ was by 25, 25, and 26%, respectively. All the parenting variables were independently associated with IQ except for the summary parenting score. A combination of Models A and B resulted in reductions of 41, 39, and 39% for the verbal, performance and full IQ measures; again, all the variables were significantly associated apart from the parenting score.

Model C involved the socio-demographic factors, and on its own accounted for reductions of 67–68% of each of the three IQ measurement differences between the children of externally oriented and internally oriented mothers. Each of the social factors had an independent effect.

Finally factors in each of Models A, B, and C were taken into account together. This resulted in a reduction in the regression coefficient from the baseline of 5.84, 4.32, and 5.75 IQ points or 80, 82, and 80%, respectively. The mediating variables that are significantly associated are shown in the final model (Supplementary Table 2).

Although the proportion of the effect size attributable to maternal externality has been similar in each of the verbal, performance and full IQ measures, both the effect size as measured by the regression coefficient and the actual variance of the IQ measures explained was much smaller for performance IQ compared to the verbal or full IQ measurements. This was true of the unadjusted measure as well as each of the final models (**Table 3**). Finally, we analyzed the data separately for boys and girls, but the results did not differ (data not shown).

**Table 3:** Summary of changes in regression coefficient and of the proportion of variance explained by the different models compared with the unadjusted model.

Model taken	Difference from	Verbal	Performance	Full
into account	unadjusted	IQ	IQ	IQ
A	*b*	+1.84	+1.48	+1.88
	*R*^2^ %	+3.46	+1.95	+3.54
B	*b*	+1.86	+1.31	+1.88
	*R*^2^ %	+5.15	+2.85	+5.40
C	*b*	+4.98	+3.58	+4.88
	*R*^2^ %	+12.47	+6.09	+12.59
A+B	*b*	+2.99	+2.04	+2.84
	*R*^2^ %	+7.62	+4.05	+7.77
A+B+C	*b*	+5.43	+3.93	+5.30
	*R*^2^ %	+13.90	+6.78	+14.23

### Effects of Mediation

Thus the mediators we have chosen have demonstrated an explanation of about 80% of the LOC association that we have shown. It is highly likely that other behaviors of the mother (or her partner) may explain the remaining 20% of the association with LOC.

### Sensitivity Analysis Using IQ at Age 4

The analysis of the IQ measure at age 4 used a much smaller sample of children, and consequently the confidence intervals are much larger than those found for the age 8 analyses. Nevertheless, there was clear evidence that maternal externality was associated with reduced full IQ even at this young age (*b* = -7.82 (95% CI -9.56, -6.09), *n* = 986, *P* < 0.0001). The final model allowing for A + B + C resulted in a reduction to *b* = -1.24 (95% CI -3.29, +0.81); *n* = 742; *P* = 0.236 (Supplementary Table 3). Although the lack of significance implies that there is no residual effect, the confidence limits of the effect size after adjustment includes the effect size shown after adjustment at age 8 (1.93).

## Discussion

### The Importance of IQ Globally

A fundamental resource of individuals is their intelligence, yet there is no universal definition of this concept. Wechsler (1944) described it as ‘the aggregate or global capacity of the individual to act purposefully, to think rationally, and to deal effectively with his environment’ but there is a myriad of other definitions (Legg and Hutter, 2007). Despite such confusion there are generally recognized measures of intelligence; the most widely recognized being the intelligence quotient (IQ) developed by Wechsler (1950).

There is evidence of high heritability (34–48%) but also evidence of *in utero* and other environmental effects (Devlin et al., 1997). Convincing evidence shows that the mean IQ of many developed populations, whether measured in infancy, childhood, or late adolescence, has increased over time (Dickens and Flynn, 2001; Lynn, 2009), thus suggesting the likely importance of environmental influences. Although there are arguments about definitions, heritability and measurement, it has become clear, irrespective of definition and test used, that data from brain-imaging and genetic studies are strongly correlated with results from intelligence tests, thus providing evidence for the validity of the IQ measurements (Deary et al., 2010).

As well as the obvious possible outcomes associated with intelligence such as academic achievement (Kalechstein and Nowicki, 1997), it is acknowledged that other broader outcomes may also be involved. For example, the financial success of nations may be tied to the abilities of their populations, which in turn are linked to their cognitive abilities. Thus, in Europe the gain of an IQ point was estimated to increase the GDP by 10,000 Euros per child in 2010 (Bierkens et al., 2012). Importantly also Veenhoven and Choi (2012) explored the relation between intelligence and happiness on two levels, at the micro-level of individuals and at the macro-level of nations. At the microlevel, they looked at the results of 23 studies and found no correlation between IQ and happiness. However, at the macro level, they assessed the correlation between average IQ and average happiness in 143 nations and found a strong positive relationship. They concluded that the findings suggest smartness of all pays more than being smarter than others.

### The Environmental Influences on IQ

Although IQ is often assumed to be an innate characteristic of a child, the strong evidence of increasing IQ levels over time suggests that there is likely to be a major environmental component to its expression. Indeed there is excellent evidence that the IQ of the population can be increased using environmental exposures. This has been illustrated for breast feeding using a cluster randomized controlled trial in Belarus (Kramer et al., 2008) which resulted in 7.5, 2.9, and 5.9 verbal, performance and full IQ point increases among the groups encouraged to breast feed.

Here we have analyzed longitudinal data collected from pregnancy onward and shown that the mother’s LOC is strongly associated with the child’s IQ such that the mothers with an external orientation tend to have children with mean IQ about 7 points (almost half a standard deviation) lower than children of mothers with an internal orientation. This difference was observed at age 4 as well as at age 8 years. Further analysis of the 8-year-old data showed that this was largely mediated by perinatal and parenting features, and that socio-demographic factors also accounted for much of the difference.

### Strengths and Weaknesses

Our data analyses may be criticized for analyzing LOC as a dichotomy rather than taking advantage of the continuous scale. We have done this because it is much easier to interpret than a small change on a continuous scale. Although we recognize that this may have masked variations that would have been informative, the fact that our results are similar when the analyses were repeated using a smaller sample, tested using a different scale on a different age group, adds validity to the findings. This subgroup of 4-year-olds showed a similar reduction in mean IQ among children of externally oriented mothers, and we were able to demonstrate that this was also largely mediated by attitudes, behaviors and achievements of external women.

However, there are a number of problems with our study: (i) we have not found a different longitudinal study that has data available to allow us to attempt to replicate these findings; (ii) the data used as mediators were all obtained from responses by the mother to questionnaires, rather than by direct observation; however, other studies have shown validation of maternal report using biomarkers in this cohort [e.g., maternal fish consumption with blood mercury and omega-3 fatty acid levels (Golding et al., 2013; Williams et al., 2001)]; (iii) we have shown that the children who had their IQ assessed were biased in terms of their social and environmental conditions. However, all of these factors were allowed for in the analyses, so bias in generalizing the longitudinal results may not be great.

One other possible disadvantage is that maternal IQ was not measured in this study. It is unclear as to what difference this may have made. Certainly there will be shared genes in common between mothers and their offspring, and it may be that certain genetic combinations will be more susceptible to environmental influences. Maternal education level could be considered a proxy for maternal IQ, but there is longitudinal evidence that a childhood external orientation often results in a failure of the individual to reach their full potential academically (Flouri, 2006).

In contrast there are a number of advantages to this study: (a) it is the largest study to date to determine the link between maternal LOC and IQ; (b) it is the only study that is prospective in nature with maternal LOC collected before the birth of the child, and consequently unaffected by the mother’s perception of the child; (c) it is the only study to have collected and analyzed many of the factors that may explain the link between maternal externality and reduced child IQ; (d) the IQ measures do not depend on report, but on direct assessment by trained psychologists unaware of the LOC of the mothers.

### The Possible Mechanisms

We have suggested that if an external mother were to become more internal, her lifestyle in regard to her offspring would change to reflect the behavior we have found associated with internal mothers (**Table 1**), i.e., she would stop smoking (or never start), would eat a healthy diet and breast feed, her parenting behavior would improve, as would some of the demographics especially if she became more internal in early adolescence (e.g., she would have obtained higher educational qualifications, delay having children until her mid-twenties, would choose a partner who was more educated and who himself had an internal LOC). If we are correct in suggesting that these are on the mediating pathway, it is important to recognize that changing a mother’s LOC to greater internality may result in consequent benefit to the child’s cognitive ability. We suggest that this possibility be subjected to rigorous evaluation through controlled research studies changing LOC as summarized elsewhere (e.g., Nowicki, 2016).

## Conclusion

We found that maternal external LOC in pregnancy is a strong indicator of lower IQ in her offspring, and that this is apparent by 4 years of age. This finding appears to be mostly associated with poor life choices made by the mother and consequent disadvantages to the child in infancy and early childhood. It would appear that efforts to increase the internality of the mother during her own childhood could generate the kinds of attitudes and behaviors that may result in an increase in the IQ of her offspring. An observational study such as this can point to such an effect, but conclusive support for these conclusions can only be obtained by a randomized controlled trial changing the LOC orientation of the female population before they become parents.

## Author Contributions

SN had the idea. JG planned the analyses. GE and SG undertook statistical analyses. SN and JG wrote the first draft. All authors contributed equally to writing later drafts, checking and editing.

## Conflict of Interest Statement

The authors declare that the research was conducted in the absence of any commercial or financial relationships that could be construed as a potential conflict of interest.
