# Supplementary material for: Prenatal Internal Locus of Control Is Positively Associated with Offspring IQ, Mediated through Parenting Behavior, Prenatal Lifestyle and Social Circumstances
Source: Front Psychol. 2017 Aug 22;8:1429. doi: 10.3389/fpsyg.2017.01429 (PMC5572283; doi:10.3389/fpsyg.2017.01429)
Supplement: Supplementary file 1 [file Table_1.pdf]

Supplementary Tables

Supplementary Table 1: Differences between the children attending the 8 year ALSPAC clinic and those who were in contact with the study but did not attend.

Demographic Factor	Attended	Did Not Attend
Sex	N=7471	N=6418
Boy	50.1%	52.9%
Girl	49.9%	47.1%
Maternal education level		
Low	21.8%	41.1
Medium	34.9	34.0
High	43.3	24.9
Maternal age		
<20	3.2	11.7
21-24	11.5	22.5
25-29	39.8	37.4
30-34	33.2	21.3
35+	12.4	7.1
Housing tenure		
Owner occupied	83.3	61.2
Council rented	9.0	24.6
Other	7.6	14.2
Ethnicity of child		
White	96.1	93.2
Non-white	3.9	6.8

Supplementary Table 2: The significant associations with all cofactors in the final model (A + B + C) concerning the relationships with the child's IQ as outcome.

Factor	Verbal IQ	Performance IQ	Full IQ
Prenatal smoking	NS	NS	NS
Prenatal alcohol	NS	NS	NS
Prenatal oily fish	NS	NS	NS
Breast fed	++++	+++	++++
Prenatal attitude	++	+++	++++
Mother reads to child	++++	NS	++
Parenting score	NS	NS	NS
Child taken to library	++++	++++	++++
Mother sings to child	+	++	++
No. of books child has	++++	++++	++++
Maternal education	++++	++++	++++
Maternal age	++++	NS	++
Lives in public housing	NS	NS	NS
Paternal occupation	++++	+++	++++
Paternal education	++++	++++	++++

Key: NS=P≥0.05; + P<0.05; ++ P<0.01; +++ P<0.001; ++++ P<0.0001

Supplementary Table 3: The unadjusted and adjusted associations between the difference between the prenatal maternal internal and external locus of control and mean IQ of the offspring at **age 4**.

Outcome and Model	b [95%CI]	n	R^2 %	P
Full-scale IQ				
Unadjusted	7.82 [6.09, 9.56]	986	7.35	<0.0001
Model A	6.68 [4.82, 8.55]	885	10.88	<0.0001
Model B	5.08 [3.24, 6.92]	844	15.11	<0.0001
Model A+B	4.55 [2.63, 6.48]	800	16.88	<0.0001
Model C	2.57 [0.65, 4.48]	861	18.71	0.009
Model A+B+C	1.24 [-0.81, 3.29]	742	23.12	0.236

Model A allows for pre- and perinatal exposures; Model B for parenting attitudes and activities; Model C for sociodemographic variables (see text for description of variables). b is the regression coefficient.
